# Like A Dew Drop On A Lotus Leaf: Perceptions Of Aging Well In South Asian American Older Adults

**DOI:** 10.1093/geroni/igab046.3622

**Published:** 2021-12-17

**Authors:** Mushira Khan, Sheetal Shah, Ajla Basic

**Affiliations:** 1 Mather Institute, Plainfield, Illinois, United States; 2 University of California (Davis), Sacramento, California, United States; 3 Mather, Evanston, Illinois, United States

## Abstract

Past research has underscored four key themes prevalent in popular and scientific discourse on successful aging in North America – the emphasis on individual agency and control; continuing productive activity into old age; the value of independence in late life; and an ideal construction of permanent personhood, wherein the realities of mortality and decline are inadequately addressed (Lamb, 2014). Yet, the meanings attached to successful aging differ across cultures and are not very well-understood. The Perceptions of Aging Well in Diverse Populations study aims to acquire a holistic understanding of the attitudes and beliefs around aging well across cultures and to identify the similarities and differences in these perceptions within diverse racial and ethnic groups. This presentation highlights preliminary findings from in-depth, semi-structured qualitative interviews with South Asian Americans 50 years and older (n=19; 9 men, 10 women). Participants shared that a sense of inevitability and aging with “grace”, “dignity”, and “wisdom” were key components of successful aging. Maintaining good health, keeping a positive attitude, and remaining independent in later life appeared motivated primarily by a desire to remain connected to, but not necessarily “burden” adult children with caregiving responsibilities. Religious faith and spiritual well-being, availability of support systems, and a sense of community were key facilitators. Limited English proficiency and loneliness posed challenges to aging well, particularly in late-life immigrants. These findings provide unique insights into subjective perceptions of successful aging and may help inform programs and policies that support the health and well-being of older South Asian Americans.

